# Single-center experience with Romiplostim N01 for the management of severe persistent cancer therapy-induced thrombocytopenia in lung cancer: Insights and outcomes

**DOI:** 10.1097/MD.0000000000051010

**Published:** 2026-10-09

**Authors:** Changjian Wang, Chuangyan Wu, Geng Wang, Kaiyue Wang, Shijiang Fei, Yan Zong, Xiaorong Dong, Sheng Zhang, Rui Zhou

**Affiliations:** a Department of Thoracic Surgery, Union Hospital, Tongji Medical College, Huazhong University of Science and Technology, Wuhan, China; b Department of Gastrointestinal Surgery, Union Hospital, Tongji Medical College, Huazhong University of Science and Technology, Wuhan, China; c Cancer Center, Union Hospital, Tongji Medical College, Huazhong University of Science and Technology, Wuhan, China; d Institute of Radiation Oncology, Union Hospital, Tongji Medical College, Huazhong University of Science and Technology, Wuhan, China; e Hubei Key Laboratory of Precision Radiation Oncology, Wuhan, China.

**Keywords:** chemotherapy-induced thrombocytopenia, real-world study, refractory thrombocytopenia, romiplostim, solid tumors, thrombopoietin receptor agonist

## Abstract

Cancer therapy-induced thrombocytopenia (CTIT) is a common complication in patients with lung cancer undergoing treatment, leading to treatment delays and dose reductions. Romiplostim N01, a thrombopoietin receptor agonist, has shown potential in managing severe persistent CTIT. This study aims to describe platelet recovery outcomes and safety associated with Romiplostim N01 for managing severe persistent CTIT in patients with lung cancer, providing real-world clinical evidence. This single-center observational study enrolled 56 patients with lung cancer who developed severe persistent CTIT and received Romiplostim N01 treatment from June 1, 2024, to February 28, 2025. Data on clinical characteristics, prior treatments, CTIT severity, and platelet recovery outcomes were retrospectively collected and analyzed. The primary endpoints were platelet recovery rates and the safety profile of Romiplostim N01. Of the 56 patients, 89.3% achieved platelet recovery to ≥100 × 10^9^/L, with a median recovery time of 16 days (range: 3–63 days). The treatment was well tolerated, with no serious adverse events. The most common adverse event was joint pain (10.7%), and thrombocytosis occurred in 26.8% of patients. No significant drug interactions or severe complications were observed. Additionally, patients received concomitant supportive therapies, such as platelet transfusions or oral thrombopoietin receptor agonists. Romiplostim N01 was associated with favorable platelet recovery proportions and a manageable safety profile in patients with lung cancer and severe persistent CTIT. These findings suggest that Romiplostim N01 is a viable option for managing severe persistent CTIT, though further large-scale, multicenter studies are needed to validate its platelet recovery outcomes and safety across diverse patient populations.

## 1. Introduction

Cancer therapy-induced thrombocytopenia (CTIT) has become a critical concern with the increasing use of novel cancer treatment modalities.^[1]^ CTIT not only impedes the delivery of planned cancer therapies but also significantly compromises patient outcomes, with persistent and refractory cases posing severe risks.^[2,3]^ The advent of targeted therapies, immunotherapies, and antibody-drug conjugates has further complicated CTIT management compared to traditional chemotherapy and radiotherapy. Studies have shown CTIT incidence exceeding 80% in patients with non-small cell lung cancer (NSCLC) receiving gemcitabine-based chemotherapy, with even higher rates in combination therapies.^[4,5]^ This highlights the urgent need for optimized CTIT management strategies to improve survival and quality of life in patients with cancer.

CTIT arises from diverse mechanisms, including bone marrow suppression, direct megakaryocyte damage, and immune-mediated platelet destruction. Chemotherapeutic agents, particularly those with myelosuppressive effects, disrupt megakaryocyte maturation and platelet release. Radiation therapy can similarly impair bone marrow function, especially when delivered to large pelvic or vertebral regions. Furthermore, certain targeted therapies and immunotherapies can provoke immune-mediated platelet destruction or interfere with thrombopoietin (TPO) signaling.^[6,7]^ Standard treatments for thrombocytopenia include platelet transfusions, corticosteroid therapy, and intravenous immunoglobulin. Platelet transfusions provide a rapid increase in platelet counts but carry risks of transfusion reactions and alloimmunization. Corticosteroids and intravenous immunoglobulin can be effective in immune-mediated thrombocytopenia but carry potential side effects and may not be suitable for all patients.^[8]^ Despite the availability of these treatments, more effective and well-tolerated options for CTIT management are still needed.

Although the National Comprehensive Cancer Network guidelines have removed recombinant human thrombopoietin (rhTPO) and recombinant human interleukin-11 (rhIL-11) from recommendations for managing CTIT due to concerns about side effects, the Chinese Society of Clinical Oncology guidelines and expert consensus in China continue to endorse treatments such as rhTPO, IL-11, and oral small-molecule TPO receptor agonists (TPO-RAs).^[9]^ TPO-RAs play a crucial role in stimulating thrombopoiesis and are increasingly used in clinical practice. Romiplostim, a novel TPO-RA, offers a promising therapeutic option for CTIT and was initially approved for the treatment of chronic immune thrombocytopenia (ITP). Romiplostim stimulates platelet production by binding to and activating the TPO receptor on megakaryocytes. Unlike endogenous TPO, which is a glycoprotein, romiplostim is a peptibody, lacking amino acid sequence homology to TPO.^[10]^ This distinction potentially minimizes the risk of neutralizing antibody formation. Subsequent clinical studies demonstrated its efficacy in treating CTIT in patients with solid tumors, leading to its approval as an adjunct therapy in oncology.^[11,12]^

Several studies have evaluated romiplostim for chemotherapy-induced thrombocytopenia. In a phase II trial, weekly romiplostim corrected platelet counts to ≥100 × 10^9^/L in 85% of treated patients within 3 weeks, allowing most to resume chemotherapy without recurrent chemotherapy-induced thrombocytopenia.^[13]^ Long-term follow-up confirmed sustained platelet recovery and safety over ≥1 year of continued romiplostim, with no evidence of resistance or excess thrombosis.^[14]^ However, specific clinical reports evaluating romiplostim’s efficacy in managing CTIT in patients with lung cancer are lacking. This study aims to evaluate the effectiveness and safety of Romiplostim N01 in patients with lung cancer and severe persistent CTIT. Romiplostim N01, independently developed by Qilu Pharmaceutical, is a second-generation long-acting TPO-RA and a biosimilar of romiplostim (Nplate). It is the first domestically developed long-acting TPO-RA peptide-fusion protein in China. Its biological structure, preclinical data, pharmacokinetics, and clinical efficacy and safety in ITP populations are consistent with those of romiplostim (Nplate). Phase III clinical trials (NCT05554913 and NCT05851027) were conducted to evaluate Romiplostim N01 for CTIT management and treatment. Due to the accessibility of Romiplostim N01, this single-center observational study used it to treat severe persistent CTIT in lung cancer, which was associated with improved platelet recovery in patients refractory to conventional approaches. Romiplostim N01 also demonstrated a favorable safety profile, with only mild adverse events (AEs).

Severe persistent CTIT is characterized by a severe decline in platelet counts <50 × 10^9^/L that remains persistently unresolved despite standard thrombopoietic interventions (e.g., platelet transfusion or rhTPO).^[15,16]^ This prolonged inadequacy frequently necessitates critical treatment delays, dose reductions, or the outright discontinuation of cancer therapies, thereby severely impacting patient survival and overall outcomes.^[2]^ Previous CTIT studies in solid tumors have not differentiated between CTIT severities or durations. This study specifically examines patients with lung cancer and severe persistent CTIT to evaluate Romiplostim N01’s efficacy and safety, providing practical clinical data. These findings support Romiplostim N01 as a rational choice for managing severe persistent CTIT in patients with lung cancer.

## 2. Methods

### 2.1. Study design and ethical approval

This observational, single-center, retrospective study screened 63 patients with lung cancer who developed severe persistent CTIT and were treated with Romiplostim N01 between June 1, 2024, and February 28, 2025, using an electronic medical record system. After screening, 56 patients received at least 2 treatment doses of Romiplostim N01 (Fig. 1). The screening process was conducted solely to assess eligibility for retrospective analysis. This study was non-interventional, meaning it did not impact patients’ treatment decisions, nor were any additional tests or procedures performed beyond standard clinical care. The research was approved by the Ethics Committee of Union Hospital, Tongji Medical College, Huazhong University of Science and Technology (approval No. 2024(0868); approval date November 4, 2024). All participants provided written informed consent for the use of their medical records in the research. Electronic medical records were accessed for research purposes on April 27, 2025. This date also served as the end of follow-up (data cutoff) and the database lock date. During the data collection period, authors had access to identifiable participant information; however, all data were anonymized for analysis.

**Figure 1.**
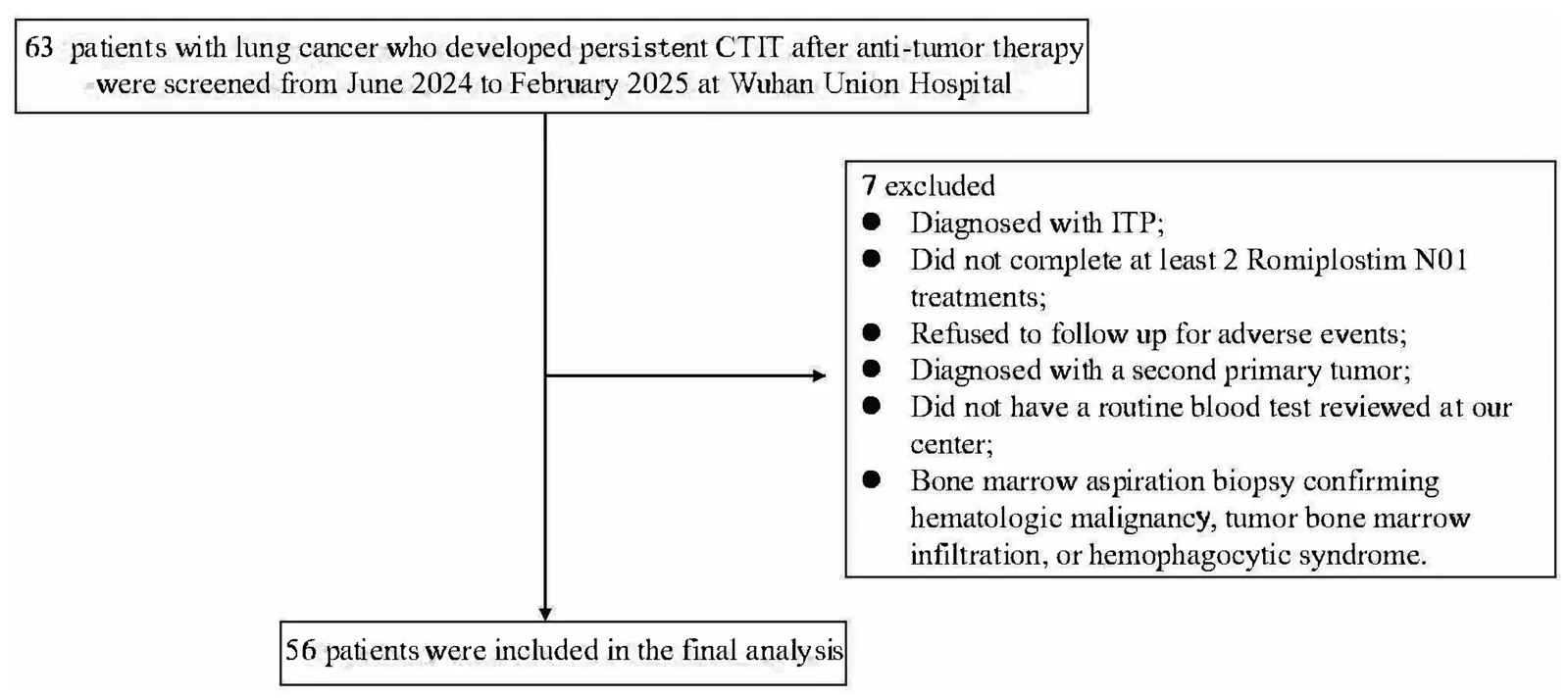
Study cohort and patient flow. CTIT = cancer therapy-induced thrombocytopenia, ITP = immune thrombocytopenia.

### 2.2. Inclusion and exclusion criteria

Based on the following inclusion criteria, patients with histologically or cytologically confirmed lung cancer diagnosed at our center between June 2024 and February 2025, an Eastern Cooperative Oncology Group performance status of 0 to 2, and severe persistent CTIT, defined as platelet counts <50 × 10^9^/L despite standard thrombopoietic interventions such as platelet transfusion, rhTPO, or corticosteroids, were included. Additionally, to ensure a highly refractory cohort, we specifically screened and enrolled patients whose severe thrombocytopenia persisted for at least 2 consecutive weeks. Patients were also required to have no prior exposure to Romiplostim N01.

Exclusion criteria were a diagnosis of ITP; receipt of fewer than 2 doses of Romiplostim N01; refusal of follow-up for AE monitoring; presence of a second primary tumor; bone marrow aspiration confirming hematologic malignancy, tumor infiltration of the bone marrow, or hemophagocytic syndrome; and incomplete follow-up blood count data in our hospital records.

### 2.3. Romiplostim N01 treatment protocol

The recommended dosing guidelines suggest an initial dose of 2 to 4 µg/kg, administered subcutaneously once weekly, with a weekly increase of 1 to 2 µg/kg, up to a maximum dose of 10 µg/kg, until platelet counts reach 100 to 150 × 10^9^/L. Dose adjustments are typically made on a weekly basis. Given that all patients in this study had refractory, severe persistent CTIT, the treatment regimen employed was 250 µg subcutaneously once per week, which corresponds to a dose range of 4 to 5 µg/kg. This decision was based on 2 main considerations: the practical accessibility of Romiplostim N01 in our center, where fixed-dose preparations are commonly used in routine clinical practice, and accumulated local clinical experience indicating that such dosing is effective and well-tolerated in refractory CTIT cases.

### 2.4. Data collection and analysis

This study was primarily descriptive, and no formal statistical comparisons between subgroups were performed due to the limited sample size and heterogeneity of treatment patterns. Patient data were retrospectively extracted from the electronic medical records. Descriptive statistics were used to summarize patient characteristics, including demographic data, baseline platelet counts, treatment regimens, severe persistent CTIT severity and duration (defined as the length of the most recent severe persistent episode prior to Romiplostim N01 initiation), and platelet recovery outcomes. Continuous variables are expressed as medians with ranges, and categorical variables as frequencies and percentages. The main software used for data visualization and analysis was GraphPad Prism version 10.0 (GraphPad Software, La Jolla). Platelet recovery rates were plotted using scatter plots and line graphs to illustrate trends in platelet counts over time. Survival analysis was considered but deemed inappropriate due to the observational nature of the study and lack of long-term follow-up data. Available data on the presence of antiplatelet antibodies were collected. Data on concomitant medications, including chemotherapy and immunotherapy agents, were collected, and potential drug interactions were assessed using available drug interaction databases. However, no formal drug interaction study was conducted. All analyses were performed using the available data, with no imputation for missing values.

## 3. Results

### 3.1. Baseline characteristics

To evaluate the use of Romiplostim N01 for managing severe persistent CTIT, this study included 56 patients with lung cancer. The cohort had a median age of 66 years (range, 60–74), with 15 females (26.8%) and 41 males (73.2%). The median body mass index was 22.66 (range, 16.71–28.69). Cancer staging, based on the ninth edition of the tumor–node–metastasis classification, classified 15 (26.8%), 22 (39.3%), and 19 (33.9%) patients into Stage IIIB, IVA, and IVB groups, respectively. The distribution by pathological types included 6 (10.7%), 34 (60.7%), and 15 (26.8%) patients with small cell carcinoma, adenocarcinoma, and squamous cell carcinoma, respectively, and 1 (1.8%) patient with other pathological types, respectively. Regarding prior anticancer treatment history, 9 (16.1%) and 47 (83.9%) patients had received monotherapy and combination regimens, respectively. The baseline platelet count before initiation of anticancer therapy was 211.5 × 10^9^/L (range, 87–229), while the nadir platelet count immediately prior to Romiplostim N01 treatment was 20.5 × 10^9^/L (range, 3–45). In addition, 19 patients (33.9%) underwent bone marrow aspiration. Notably, the median duration of the most recent severe persistent CTIT episode prior to Romiplostim N01 initiation was 27 days (range, 14–42). For supportive therapy to recover severe persistent CTIT prior to romiplostim initiation, 47 patients (83.9%) received platelet transfusions, 56 (100%) received rhTPO, 11 (19.6%) received rhIL-11, 43 (76.8%) received oral TPO-RAs (excluding romiplostim), and 30 (53.6%) received corticosteroids (Table 1).

**Table 1:** Baseline demographic, oncological, and CTIT-related characteristics of patients with severe persistent CTIT receiving Romiplostim N01.

Characteristic	Value
Age, yr	66 (60–74)
Female:Male, n:n	15:41
BMI	22.66 (16.71–28.69)
Cancer stage, n (%) (TNM classification)	
IIIB	15 (26.8)
IVA	22 (39.3)
IVB	19 (33.9)
Pathological type, n (%)	
Small cell carcinoma	6 (10.7)
Adenocarcinoma	34 (60.7)
Squamous cell carcinoma	15 (26.8)
Others	1 (1.8)
Antitumor therapy, n (%)	
Monotherapy	9 (16.1)
Combination therapy	47 (83.9)
Baseline platelet before anticancer therapy (×10^9^/L), median (range)	211.5 (87–229)
Nadir platelet prior to Romiplostim N01 (×10^9^/L), median (range)	20.5 (3–45)
Patients undergoing bone marrow aspiration, n (%)	19 (33.9)
Patients accepting combination therapies to recover CTIT, n (%)	
Platelets transfusion	47 (83.9)
rhTPO	56 (100)
rhIL-11	11 (19.6)
TPO-RA (exclude romiplostim)	43 (76.8)
Corticosteroids	30 (53.6)
CTIT duration, d	27 (14–42)

Values are n/N (%), median (range), or mean ± SD as appropriate. No formal hypothesis testing was performed. Duration refers to the most recent persistent episode prior to Romiplostim N01 initiation.

BMI = body mass index, CTIT = cancer therapy-induced thrombocytopenia, rhTPO = recombinant human thrombopoietin, SD = standard deviation, TNM = tumor–node–metastasis, TPO-RA = thrombopoietin receptor agonist.

### 3.2. Treatment course and efficacy outcomes

Platelet counts were assessed before, during, and after treatment with Romiplostim N01 to evaluate its efficacy in managing severe persistent CTIT (Table 2). Prior to treatment with Romiplostim N01, patients experienced a significant decline in platelet counts, with a median nadir of 20.5 (×10^9^/L) (range, 3–45 × 10^9^/L). Following treatment, 50 patients (89.3%, 95% confidence interval [CI]: 78.5%–95.0%) achieved platelet recovery to ≥100 × 10^9^/L, with a median recovery time of 16 days (95% CI: 12–21 days; range, 3–63 days). In terms of clinical recovery thresholds, a platelet count of ≥50 × 10^9^/L was achieved in 55 out of 56 patients (98.2%, 95% CI: 90.6%–99.7%), and a platelet count of ≥75 × 10^9^/L was achieved in 52 out of 56 patients (92.9%, 95% CI: 83.0%–97.2%). In addition to Romiplostim N01, all patients had prior or concomitant exposure to at least 1 supportive therapy, including 22 (39.3%) who received platelet transfusions, 3 (5.4%) who received rhTPO, 6 (10.7%) who received rhIL-11, 28 (50.0%) who received oral small-molecule TPO-RAs, and 19 (33.9%) who were treated with corticosteroids (Fig. 2A). The detailed overlapping and combination patterns of these concurrent supportive therapies are further illustrated in the Venn diagram (Fig. 2C). Following platelet recovery, 52 patients (92.9%) were able to successfully resume subsequent anticancer therapy. Among these, 32 patients (61.5% of the resumed cohort) maintained their original chemotherapy dose intensity, whereas 20 patients (38.5%) required a dosage reduction due to clinical indications (Fig. 2B). Although most patients exhibited platelet recovery during Romiplostim N01 treatment, a few did not achieve the expected platelet recovery.

**Table 2:** Treatment outcomes in patients with severe persistent CTIT receiving Romiplostim N01.

Characteristic	Value
Platelet recovery (≥100 × 10^9^/L), n (%)	50 (89.3)*
Platelet recovery (≥75 × 10^9^/L), n (%)	52 (92.9)†
Platelet recovery (≥50 × 10^9^/L), n (%)	55 (98.2)‡
Time to recovery (≥100 × 10^9^/L), d, median (range)	16 (3–63)§
Concomitant therapies after Romiplostim N01 initiation, n (%)	
Platelets transfusion	22 (39.3)
rhTPO	3 (5.4)
rhIL-11	6 (10.7)
TPO-RA (exclude romiplostim)	28 (50.0)
Corticosteroids	19 (33.9)
Continuation of cancer therapy after recovery, n (%)	52 (92.9)
Required dose reduction	20 (38.5)

CI = confidence interval, CTIT = cancer therapy-induced thrombocytopenia, rhTPO = recombinant human thrombopoietin, TPO-RA = thrombopoietin receptor agonist.

* 95% CI: 78.5% to 95.0%.

† 95% CI: 83.0% to 97.2%.

‡ 95% CI: 90.6% to 99.7% (CIs for proportions were calculated using the Wilson score method).

§ 95% CI: 12 to 21 days for the median recovery time.

**Figure 2.**
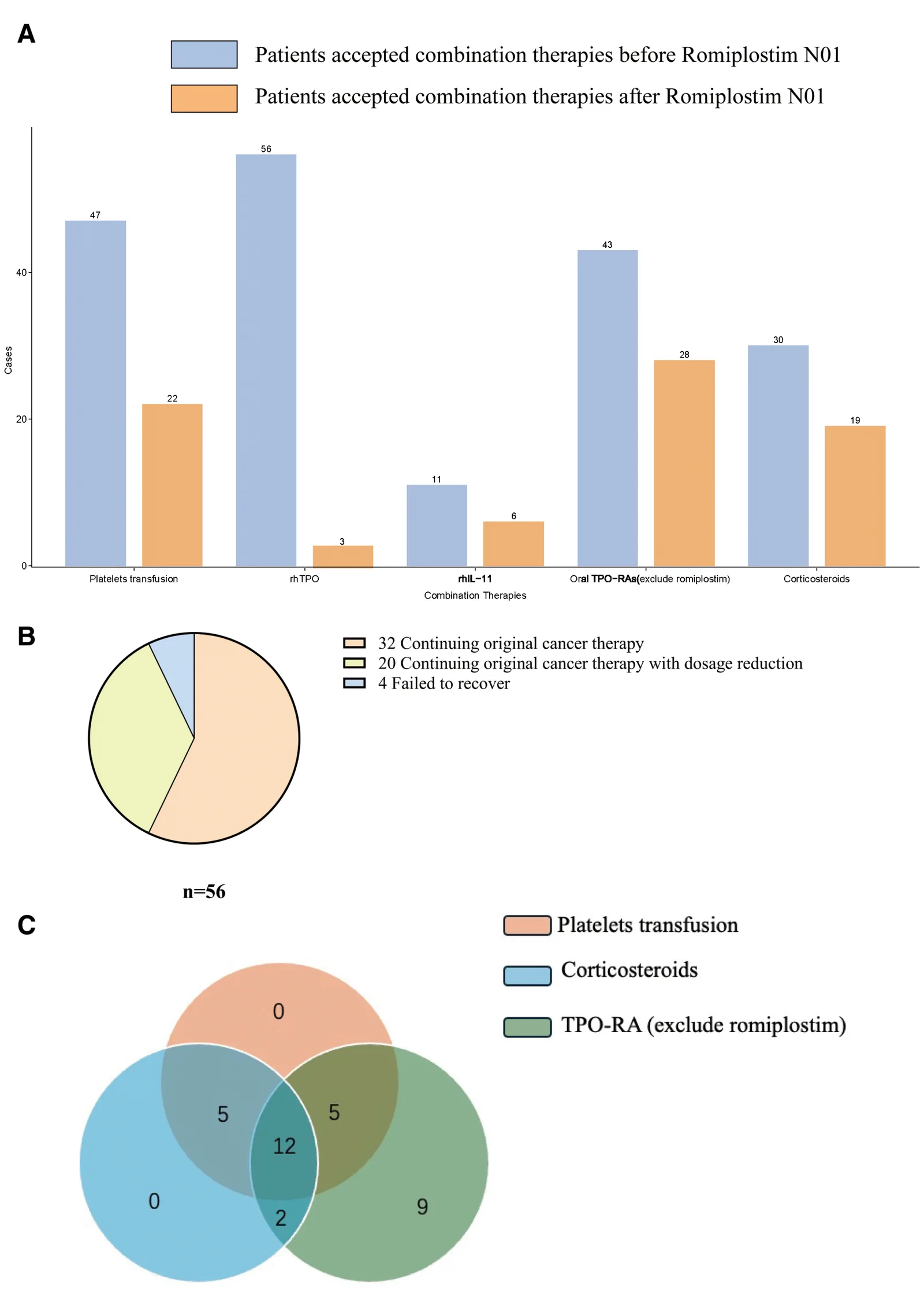
Therapeutic effects of Romiplostim N01. (A) Concurrent supportive therapies used for CTIT management before (left) and after (right) Romiplostim N01 initiation. (B) Continuation of anticancer therapy following platelet recovery: most patients (52/56, 92.9%) were able to resume treatment, although 20 (38.5%) required dose reductions. (C) Venn diagram illustrating overlap of 3 representative supportive therapies (platelet transfusions, oral TPO-RAs, and corticosteroids) administered after Romiplostim N01 initiation. Only these 3 categories are shown for clarity; therefore, the numbers do not sum to the full cohort size of 56. CTIT = cancer therapy-induced thrombocytopenia, rhIL-11 = recombinant human interleukin-11, rhTPO = recombinant human thrombopoietin, TPO-RA = thrombopoietin receptor agonist.

### 3.3. Safety and tolerability

The safety and tolerability of Romiplostim N01 were assessed in 56 patients who completed at least 2 treatment doses. Overall, the treatment was well tolerated, with no serious AEs reported. Mild AEs were observed in a small number of patients, including headache in 4 (7.1%) and joint pain in 6 (10.7%). Thrombocytosis occurred in 15 patients (26.8%), but in all cases, it was transient and resolved spontaneously or after dose adjustment. Importantly, no venous thromboembolism, arrhythmia, weight gain, pyrexia, or other serious complications occurred, and none of the patients discontinued treatment due to AEs (Table 3). These findings indicate that Romiplostim N01 has a favorable safety profile in patients with lung cancer and severe persistent CTIT, although the limited follow-up duration precludes definitive assessment of long-term thrombotic risk.

**Table 3:** Adverse events and safety profiles in patients with severe persistent CTIT receiving Romiplostim N01.

Characteristic	Value
Adverse events, n (%)	
Headache	4 (7.1)
Joint pain	6 (10.7)
Thrombocytosis	15 (26.8)
Arrhythmia	0
Weight gain	0
Pyrexia	0
Serious adverse events (SAEs), n (%)	0

Adverse events (AEs) are presented as n (%). Thrombocytosis was defined as a platelet count ≥450 × 10^9^/L. Severity was graded per CTCAE v5.0. Drug-relatedness was adjudicated by investigators. No thromboembolic events occurred during the observation window.

AE = adverse event, CTCAE = Common Terminology Criteria for Adverse Events, CTIT = cancer therapy-induced thrombocytopenia.

### 3.4. Case examples

To further illustrate the impact of Romiplostim N01 on platelet recovery and its use in combination with other therapies, we present 3 specific cases. These cases further illustrate the effectiveness of Romiplostim N01 in managing severe persistent CTIT in patients with lung cancer undergoing oncology treatment. Patients received combination therapies before and after Romiplostim N01.

#### 3.4.1. Case 1

A 63-year-old male with metastatic lung adenocarcinoma (cT4N3M1c) presented with persistent grade IV thrombocytopenia. His initial platelet count was 225 × 10^9^/L. He had received multiple lines of chemotherapy and immunotherapy, culminating in albumin-bound paclitaxel and cisplatin with pembrolizumab and bevacizumab. After 2 cycles, he developed severe thrombocytopenia. Bone marrow biopsy ruled out hematologic malignancies (Fig. 3B). Initial management with rhTPO, avatrombopag, and platelet transfusions was ineffective. After 5 months of severe persistent CTIT, Romiplostim N01 (5 μg/kg, once weekly) was initiated alongside avatrombopag. Following the first dose, his platelet count significantly increased, eliminating the need for further transfusions. By day 28, his platelet count exceeded 200 × 10^9^/L, allowing discontinuation of platelet-boosting therapies. He resumed chemotherapy with the upgraded immunotherapy regimen and has not experienced CTIT recurrence (Fig. 3A).

**Figure 3.**
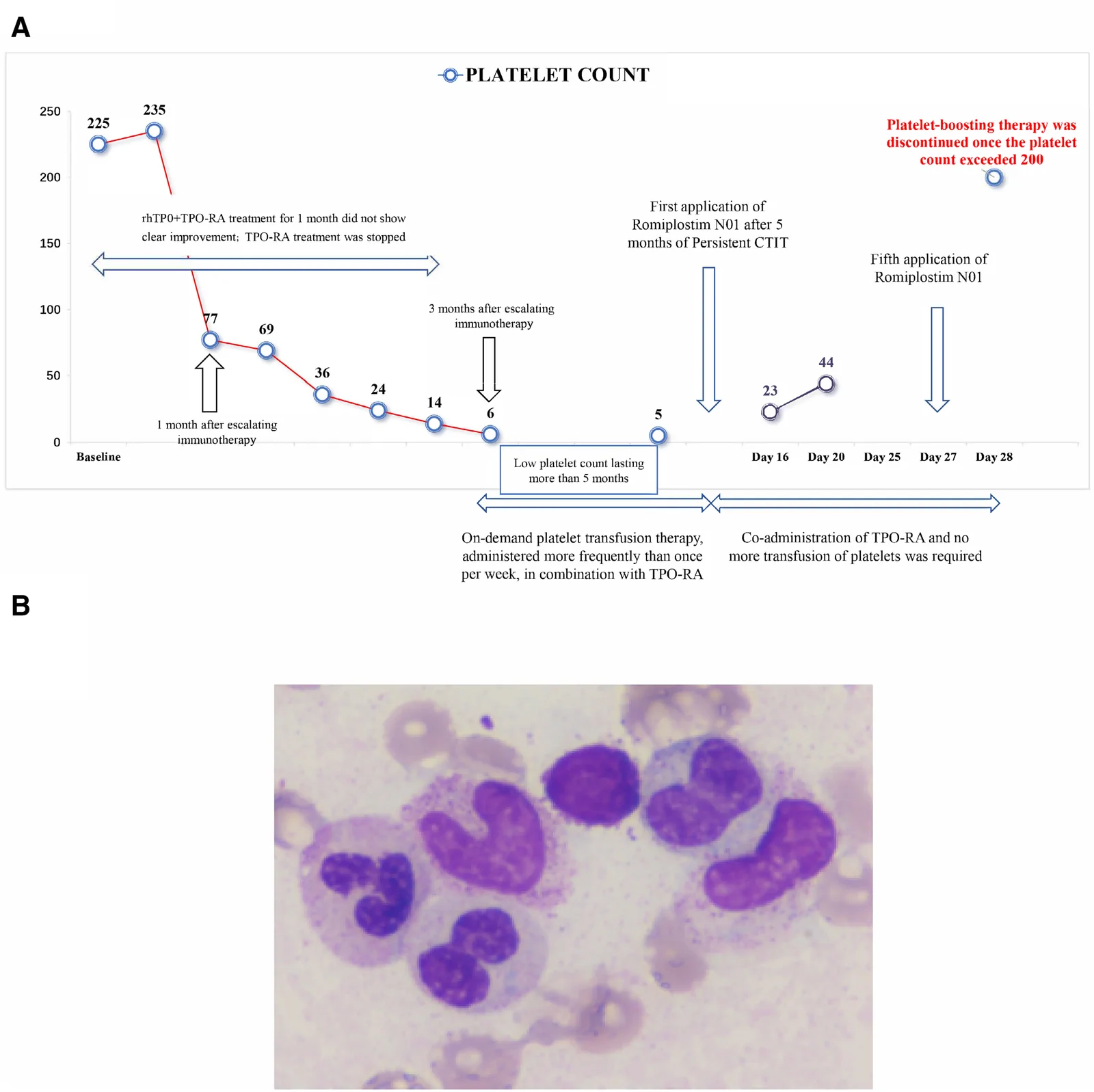
Representative case 1: clinical timeline and laboratory response. (A) Line graph depicting the patient’s platelet count (×10^9^/L) over time (days). Key treatment milestones are indicated: initiation of intensified immunotherapy, onset of grade IV thrombocytopenia, commencement of Romiplostim N01 (5 μg/kg weekly), and cessation of platelet-boosting therapies. Note the rapid platelet count increase following Romiplostim N01 administration. (B) Bone marrow biopsy micrograph. Representative image of the bone marrow biopsy, demonstrating the absence of hematologic malignancy. CTIT = cancer therapy-induced thrombocytopenia, TPO-RA = thrombopoietin receptor agonist.

#### 3.4.2. Case 2

A 62-year-old male with Stage IIIB small cell lung cancer (cT3N2aM0) developed thrombocytopenia (nadir 37 × 10^9^/L) after 1 cycle of etoposide and cisplatin. His baseline platelet count was 219 × 10^9^/L. Baseline axial chest computed tomography is shown (Fig. 4A). Treatments with rhTPO, hetrombopag, and platelet transfusions were initiated. His platelet count remained below 50 × 10^9^/L for 21 days post-chemotherapy, fulfilling the criteria for severe persistent CTIT. He developed conjunctival edema and eversion of his right eye due to thrombocytopenia and bleeding (Fig. 4C). On day 21, Romiplostim N01 (5 μg/kg) was added to hetrombopag therapy. Following Romiplostim N01 initiation, platelet transfusions were no longer needed. By day 6 post-initiation (Day 27 of the clinical course), his platelet count reached 212 × 10^9^/L (Fig. 4B), and he was able to resume cancer treatment at a reduced dose. The conjunctival edema and eversion resolved (Fig. 4D). Thrombocytosis (peak 950 × 10^9^/L) occurred after the third weekly dose of Romiplostim N01 (not shown in Fig. 4B as it occurred after the Day 28 observation window). This was considered drug-related; however, no thrombotic events were observed, and this transient increase resolved smoothly after subsequent dose adjustment, with platelet counts gradually decreasing to 383 × 10^9^/L without clinical sequelae. The conjunctival edema and eversion were likely multifactorial, potentially related to both thrombocytopenia- and chemotherapy-induced vascular fragility. While thrombocytopenia contributed to bleeding, chemotherapy may have exacerbated the condition.

**Figure 4.**
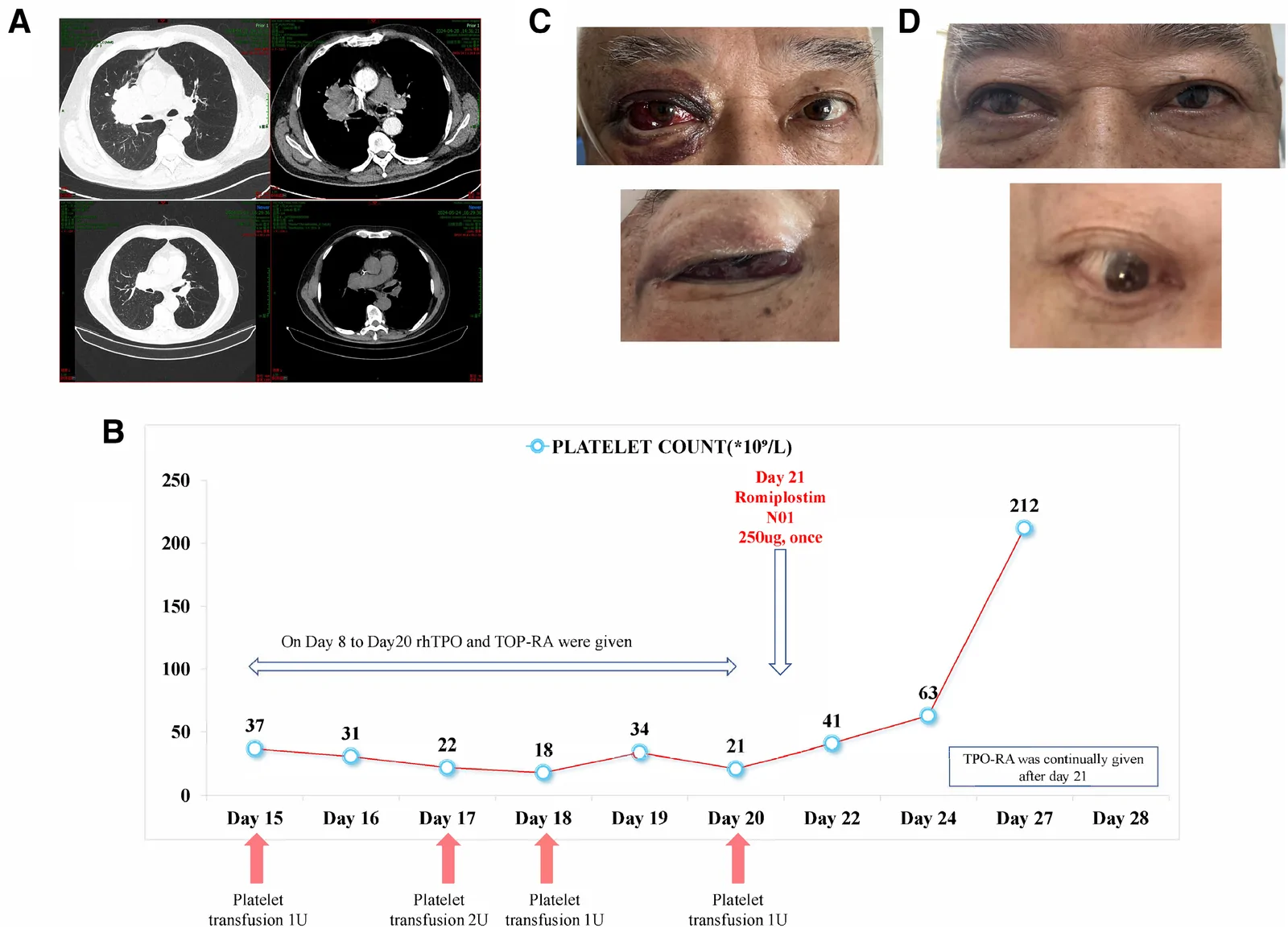
Representative case 2: CT imaging, platelet trajectory, and ocular findings. (A) Chest CT scans: Comparative CT images showing the patient’s chest before (top panel) and after (bottom panel) 1 cycle of etoposide and cisplatin chemotherapy, demonstrating a partial response. (B) Platelet count dynamics: Line graph illustrating the patient’s platelet count (×10^9^/L) over time (days). The graph highlights the initial thrombocytopenia post-chemotherapy, the period of persistent low platelet counts despite rhTPO, hetrombopag, and platelet transfusions, and the marked increase following Romiplostim N01 (250 μg on day 21) initiation. Key interventions are noted. (C) Pretreatment ocular presentation: Photographs showing conjunctival edema and eversion of the right eye, attributed to thrombocytopenia and associated bleeding, prior to Romiplostim N01 treatment. (D) Posttreatment ocular resolution: Photographs demonstrating significant resolution of conjunctival edema and eversion of the right eye following platelet recovery with Romiplostim N01. CT = computed tomography, rhTPO = recombinant human thrombopoietin, TPO-RA = thrombopoietin receptor agonist.

#### 3.4.3. Case 3

A 65-year-old female with metastatic lung adenocarcinoma (cT4N3M1a) harboring an rearranged during transfection fusion mutation experienced an overall 10-month course of thrombocytopenia, during which she developed multiple episodes of severe persistent CTIT. Her baseline platelet count was 212 × 10^9^/L. She initially received pemetrexed and cisplatin chemotherapy, followed by rearranged during transfection-targeted therapy. After 10 cycles of targeted therapy, recurrent thrombocytopenia developed, with a nadir of 7 × 10^9^/L, necessitating treatment interruption. Multiple supportive interventions were attempted, including rhIL-11, rhTPO, avatrombopag, hetrombopag, immunoglobulin, and corticosteroids, but platelet counts remained suboptimal. Romiplostim N01 was then initiated, and after 3 doses, her platelet count rose above 100 × 10^9^/L, permitting discontinuation of all other supportive therapies (Fig. 5). She subsequently resumed targeted therapy without complications. This case underscores the efficacy of Romiplostim N01 in reversing long-standing thrombocytopenia with recurrent severe persistent CTIT episodes in the setting of complex oncologic treatment.

**Figure 5.**
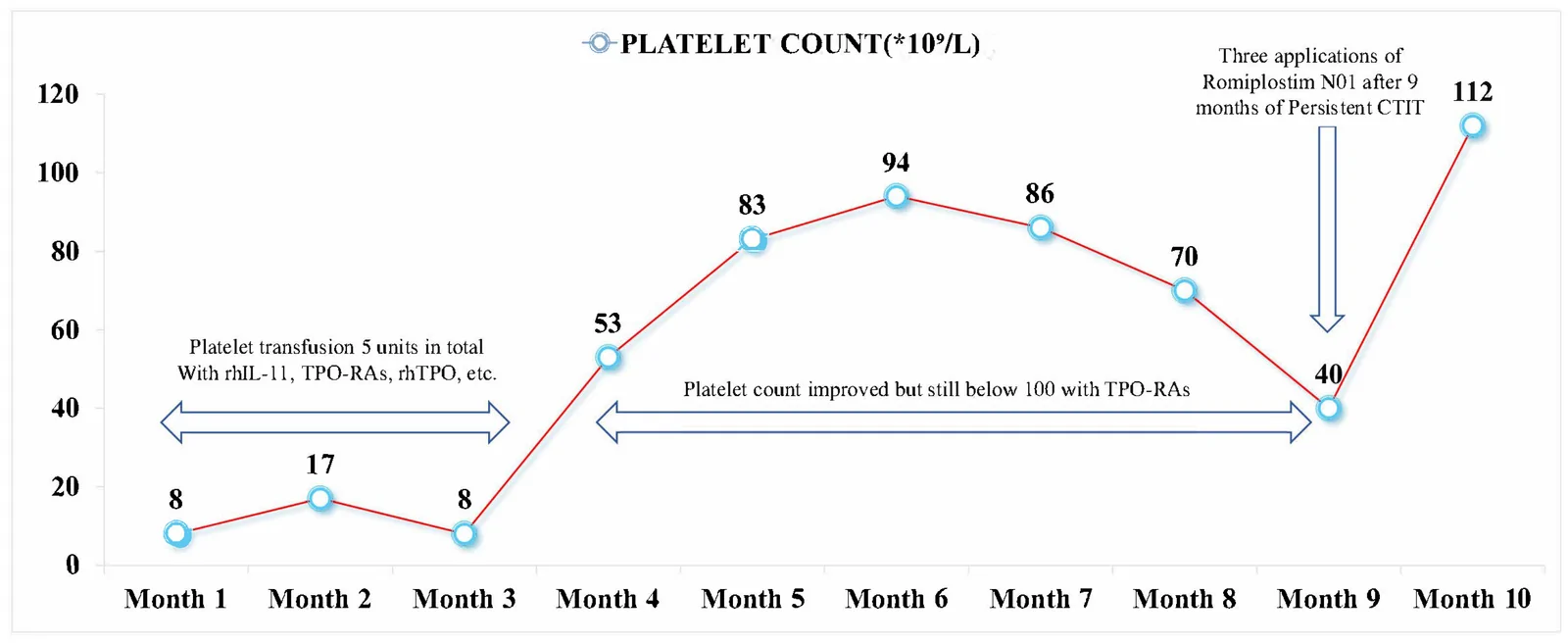
Case 3: Romiplostim N01 efficacy in prolonged refractory thrombocytopenia. Line graph illustrating the platelet count (×10^9^/L) trajectory of a 65-year-old female with metastatic lung adenocarcinoma and persistent CTIT. The graph highlights the patient’s prolonged thrombocytopenia (9 months) despite multiple platelet-boosting therapies (platelet transfusions, rhIL-11, rhTPO, avatrombopag, hetrombopag, immunoglobulin, and corticosteroids) and the significant increase in platelet count following Romiplostim N01 initiation (three doses over 3 weeks). CTIT = cancer therapy-induced thrombocytopenia, rhIL-11 = recombinant human interleukin-11, rhTPO = recombinant human thrombopoietin, TPO-RA = thrombopoietin receptor agonist.

## 4. Discussion

Severe persistent CTIT frequently necessitates treatment delays, dose reductions, or outright interruptions, significantly impacting patient outcomes. Studies have documented that in patients with advanced NSCLC, breast cancer, and ovarian cancer, 32%, 33%, and 44%, respectively, experienced treatment delays of 7 days or more due to CTIT. Similarly, substantial proportions of these patients required dose reductions of 15% or more.^[17,18]^ These dose adjustments can be quantified using relative dose intensity (RDI), a metric representing the percentage of chemotherapy dose administered relative to the standard dose for a given regimen. Reductions in RDI, driven by dose reductions and treatment delays, are critical determinants of treatment efficacy. A retrospective analysis of 3866 patients with advanced NSCLC demonstrated that dose delays of 7 days or more, or an RDI below 85%, were significantly associated with decreased survival rates.^[17]^ Furthermore, a systematic review across metastatic solid tumors corroborated that achieving an RDI of 85% or higher correlated with improved progression-free and overall survival.^[19]^ Consequently, the current paradigm in CTIT management emphasizes the implementation of safe and effective interventions to mitigate RDI reductions, thereby optimizing patient prognosis.^[18,20]^

Initial clinical investigations into CTIT management focused on hematopoietic agents, including rhIL-11, rhTPO, and recombinant human megakaryocyte growth and development factor (PEG-rHuMGDF).^[21–23]^ Oprelvekin (rhIL-11, Neumega), while initially approved for CTIT, was eventually discontinued due to modest efficacy and significant side effects.^[24]^ Although rhTPO and PEG-rHuMGDF showed promise in early trials, the development of neutralizing antibodies against PEG-rHuMGDF, which cross-reacted with endogenous TPO, led to its discontinuation in Western countries.^[25–27]^ Despite these setbacks, rhTPO and rhIL-11 remain within the Chinese Society of Clinical Oncology guidelines for CTIT management.

Second-generation TPO-RAs have emerged as pivotal agents in CTIT management. Among these, romiplostim, a subcutaneous peptibody, and oral small-molecule drugs such as eltrombopag, avatrombopag, lusutrombopag, and hetrombopag have gained regulatory approval for various thrombocytopenic conditions.^[28–32]^ Notably, hetrombopag is approved in China for CTIT treatment. Qin et al^[33]^ demonstrated that hetrombopag accelerated platelet recovery in patients with advanced solid tumors, achieving a median time to platelet count of 100 × 10^9^/L or more in 7.5 days. However, clinical data on lusutrombopag for CTIT remain sparse, and eltrombopag is primarily investigated for CTIT management.

Romiplostim’s distinctive mechanism of action, particularly its more effective activation of multiple signaling pathways, including the JAK/STAT3 pathway, compared with other TPO-RAs, highlights its significant therapeutic potential.^[34,35]^ Its clinical advantages are further amplified by its convenient once-weekly administration, the absence of dietary restrictions, and its lack of homology with endogenous TPO, minimizing the risk of neutralizing antibody formation.^[36,37]^ Romiplostim’s pharmacokinetic profile is characterized by its internalization via the neonatal Fc receptor on endothelial cells, which shields it from degradation and facilitates recycling back into circulation, yielding an extended half-life of 120 to 140 hours. Clearance occurs through the reticuloendothelial system, independent of hepatic or renal function.^[38]^ This aligns with clinical observations demonstrating a favorable hepatic safety profile across all clinical studies in patients with ITP, where romiplostim exhibited no liver-related AEs.^[39]^ Furthermore, its low propensity for drug–drug interactions enhances its suitability for patients with comorbidities.^[40]^ Notably, romiplostim presents a low risk of thrombosis.^[41]^ The predominant side effects, headache and joint pain, contribute to its overall favorable safety and tolerability profile compared to alternative CTIT treatments (Table 3).

In our study, transient thrombocytosis (platelet overshoot) occurred in 15 patients (26.8%), representing a notable rate of elevated platelet counts. However, no symptomatic venous or arterial thromboembolic events were observed during the study window. This favorable safety outcome is consistent with larger clinical trials and real-world cohorts evaluating romiplostim in solid tumors, such as the multicenter study by Al-Samkari et al,^[12]^ which demonstrated that TPO-RA-induced transient thrombocytosis does not significantly increase the background risk of thromboembolism in cancer patients undergoing active chemotherapy. This safety profile is likely attributed to the transient nature of the overshoot, which was actively managed clinically by withholding or reducing Romiplostim N01 doses once counts exceeded safety thresholds, as well as the physiological fact that TPO-RA-stimulated platelets lack the pathological hyper-activation seen in clonal myeloproliferative disorders.

The management of severe persistent CTIT often necessitates combination therapies, a practice well-reflected in our clinical experience. Recognizing the complex interplay of factors contributing to thrombocytopenia in patients with cancer, a multifaceted approach is frequently warranted. While rhTPO and romiplostim, both targeting the c-Mpl receptor, are mechanistically incompatible for concurrent use due to potential competitive inhibition, the synergistic potential of oral TPO-RAs and corticosteroids alongside romiplostim is supported by clinical guidelines.^[42]^ This approach allows for the exploitation of distinct mechanisms of action, maximizing platelet production while mitigating potential adverse effects. Furthermore, the sequential use of TPO-RAs emerges as a valuable strategy in cases of treatment failure or intolerance.^[43]^ Given the variability in patient responses and drug tolerability, the ability to transition between TPO-RAs offers a degree of flexibility crucial in optimizing treatment outcomes. This approach acknowledges the dynamic nature of CTIT and the need for personalized therapeutic strategies. The rationale behind combination therapies extends beyond mere platelet count elevation. It encompasses the broader goal of maintaining optimal RDI of cancer treatments, thereby preserving treatment efficacy and improving patient prognosis. By strategically combining agents with complementary mechanisms of action, clinicians can potentially minimize treatment interruptions, reduce dose reductions, and ultimately enhance patient survival. We acknowledge that all patients in our study received combination therapies, which makes it challenging to isolate the specific contribution of Romiplostim N01 to platelet recovery. In this observational study, our aim was to reflect real-world clinical practice, where combination therapies are commonly used to manage severe persistent CTIT. Therefore, we did not attempt to distinguish the independent effects of Romiplostim N01 from other treatments. This single-center retrospective design with frequent concomitant therapies (rhTPO, oral TPO-RAs, platelet transfusion, and/or corticosteroids) precludes causal attribution; our analyses describe associations with observed platelet recovery rates rather than treatment effects. Future studies should employ randomized controlled trial designs to isolate the independent effects of Romiplostim N01. Interpreting our findings within the context of combination therapies, further research is needed to evaluate the independent efficacy of Romiplostim N01. Similarly, due to the observational nature of our study and the use of multiple concurrent therapies in most patients, isolating the specific effects of Romiplostim N01 on liver function and other hematological and inflammatory parameters was challenging. Therefore, our primary focus was on platelet count recovery. However, the implementation of combination therapies necessitates careful consideration of potential drug–drug interactions, overlapping toxicities, and the overall treatment burden. Future research should focus on elucidating the optimal combination strategies, identifying predictive biomarkers for treatment response, and developing personalized algorithms for CTIT management.

Our findings align with previous studies demonstrating romiplostim’s efficacy in CTIT management. Soff et al^[11]^ reported an 85% platelet response rate in patients with solid tumors who were treated with romiplostim. Similarly, a retrospective cohort study by Al-Samkari et al observed a 71% response rate and a reduction in treatment delays and platelet transfusions.^[12]^ Our study extends these findings by focusing specifically on patients with lung cancer and severe persistent CTIT.

However, its retrospective design, small sample size, and heterogeneous patient population (including different cancer stages, prior treatments, and distinct biological subtypes such as NSCLC and small cell lung cancer) limit the scope for subgroup and comparative analyses. The small sample size also limits the statistical power, increasing the risk of false-positive or false-negative results, particularly when evaluating treatment efficacy. Additionally, the estimated effect size may be biased, potentially overestimating or underestimating the actual therapeutic effect of Romiplostim N01. In addition, this study lacked a control group, precluding direct comparison with other thrombopoietic agents. We plan to include patients who received rhTPO or IL-11 alone during the same period in future analyses. Another limitation is that Romiplostim N01 was never used as a monotherapy; most patients received at least 1 additional supportive therapy (e.g., platelet transfusions, rhTPO, rhIL-11, oral TPO-RAs, or corticosteroids), either before or during romiplostim treatment. This makes it difficult to attribute the efficacy exclusively to Romiplostim N01, and the potential cumulative effects of prior supportive care cannot be completely ruled out. This clinical pattern was heavily influenced by drug accessibility and pharmacoeconomic considerations at our center during the study period. When Romiplostim N01 first became available, its acquisition cost was considerably higher than that of established first-line thrombopoietic agents, such as rhTPO or rhIL-11. Consequently, Romiplostim N01 was primarily reserved as a salvage or sequential therapy for patients who had already demonstrated an inadequate response to standard interventions, thereby inherently shaping our cohort into a predominantly severe, refractory population with extensive prior or concomitant treatments. Nevertheless, this pattern reflects real-world practice, in which thrombopoietic agents are rarely administered in isolation, and the consistently high platelet recovery rate suggests that romiplostim played a central role in platelet restoration. The short follow-up duration also precluded evaluation of sustained platelet recovery, relapse rates, and long-term safety outcomes such as thrombotic risk. In addition, a fixed-dose regimen was used, which differs from the guideline-recommended stepwise escalation strategy and may limit comparability with other studies. Future large-scale, prospective, multicenter studies are warranted to validate these findings and explore the role of Romiplostim N01 in the management of CTIT. Future studies should also focus on evaluating its long-term safety profile, particularly thrombotic risks, and its efficacy in combination with other thrombopoietic agents across different patient populations. Furthermore, due to the retrospective nature of the study, granular patient-level data regarding the exact daily duration of transient asymptomatic thrombocytosis and individual day-to-day dose titration steps were not fully captured in the electronic medical records. Additionally, not all patients had undergone testing for antiplatelet antibodies, and pharmacokinetic data were also not routinely collected. These limitations are therefore acknowledged. Despite these limitations, this study provides valuable real-world data for clinical decision-making in managing severe persistent CTIT in patients with lung cancer. While Romiplostim N01 was associated with favorable recovery in our study, its primary use in China limits the international generalizability of our findings. Future studies involving diverse patient populations across multiple geographic locations are needed to validate these results and establish Romiplostim N01’s broader applicability in managing severe persistent CTIT.

Our study demonstrated that Romiplostim N01 was associated with platelet recovery in most patients with severe persistent CTIT. However, we observed that patients responded to the treatment differently. This variation likely arose from a combination of factors. These include the initial severity of the patient’s condition, other medications they were receiving, and possibly their genetic makeup. For example, patients with lower initial platelet counts or more severe conditions may need a longer treatment duration to reach the desired platelet levels. Also, other medications, such as chemotherapy or immunotherapy, may interact with Romiplostim N01 and affect its effectiveness. In the 4 patients who did not achieve platelet recovery, possible reasons included insufficient treatment duration, advanced tumor stage, or impaired bone marrow function. To better understand why patients respond differently to Romiplostim N01, future studies should systematically evaluate the following potential factors: differences in baseline platelet counts and CTIT severity, the impact of concomitant medications and other treatments, and potential genetic or epigenetic factors influencing drug response. These studies should involve larger groups of patients, use control groups for comparison, and determine the best ways to use Romiplostim N01 for different patient groups.

In our study, descriptive comparisons suggested higher observed platelet recovery proportions with Romiplostim N01 relative to concomitantly used rhTPO and oral TPO-RAs. The safety profile appeared acceptable, with AEs infrequent and no serious events recorded, and the weekly dosing regimen was practically convenient. By supporting platelet recovery, Romiplostim N01 may help enable timely resumption of anticancer therapy; prospective controlled studies are required to determine any effects on clinical outcomes.

## 5. Conclusion

In this single-center retrospective cohort, we described real-world platelet recovery outcomes associated with Romiplostim N01 use in patients with lung cancer and severe persistent CTIT. In our cohort, 89.3% of patients achieved platelet counts ≥100 × 10^9^/L, with a median time to recovery of 16 days (range, 3–63 days). Exploratory subgroup observations suggested higher recovery rates among patients unresponsive to prior therapies; however, these nonrandomized data preclude causal inference. Romiplostim N01 was generally well tolerated, and AEs were infrequent; no serious AEs were recorded. While encouraging, the retrospective design, frequent concomitant therapies, and limited sample size warrant cautious interpretation. Prospective, adequately powered controlled studies are needed to validate these associations and to delineate the potential role of Romiplostim N01 in the management of CTIT in lung cancer and other malignancies. Additionally, exploring optimal combination strategies with rhTPO, oral TPO-RAs, platelet transfusion, and/or corticosteroids in prospective controlled designs would be informative.

## Acknowledgments

We thank Dr Lin for technical support in data processing. We appreciate the constructive comments from the anonymous reviewers.

## Author contributions

**Conceptualization:** Sheng Zhang, Rui Zhou.

**Data curation:** Chuangyan Wu.

**Formal analysis:** Shijiang Fei.

**Funding acquisition:** Chuangyan Wu, Rui Zhou.

**Investigation:** Yan Zong.

**Methodology:** Geng Wang.

**Project administration:** Sheng Zhang.

**Resources:** Xiaorong Dong.

**Software:** Kaiyue Wang.

**Validation:** Chuangyan Wu, Sheng Zhang, Rui Zhou.

**Writing – original draft:** Changjian Wang.

**Writing – review & editing:** Changjian Wang.

## References

[1] Luz JH Luz PM Marchiori E. Partial splenic embolization to permit continuation of systemic chemotherapy. Cancer Med. 2016;5:2715–20. 10.1002/cam4.856 PMC5083724 27611010

[2] Al-Samkari H Soff GA. Clinical challenges and promising therapies for chemotherapy-induced thrombocytopenia. Expert Rev Hematol. 2021;14:437–48. 10.1080/17474086.2021.1924053 33926362

[3] Weycker D Hatfield M Grossman A. Risk and consequences of chemotherapy-induced thrombocytopenia in US clinical practice. BMC Cancer. 2019;19:151. 10.1186/s12885-019-5354-5 PMC6376753 30764783

[4] de Jong C Herder GJM Deneer VHM. Genetic variants as predictors of toxicity and response in patients with non-small cell lung cancer undergoing first-line platinum-based chemotherapy: design of the multicenter PGxLUNG study. Thorac Cancer. 2020;11:3634–40. 10.1111/1759-7714.13683 PMC7705630 33073546

[5] Shaw JL Nielson CM Park JK Marongiu A Soff GA. The incidence of thrombocytopenia in adult patients receiving chemotherapy for solid tumors or hematologic malignancies. Eur J Haematol. 2021;106:662–72. 10.1111/ejh.13595 PMC8248430 33544940

[6] Lonial S Waller EK Richardson PG. Risk factors and kinetics of thrombocytopenia associated with bortezomib for relapsed, refractory multiple myeloma. Blood. 2005;106:3777–84. 10.1182/blood-2005-03-1173 PMC1895114 16099887

[7] Zhang H Nimmer PM Tahir SK. Bcl-2 family proteins are essential for platelet survival. Cell Death Differ. 2007;14:943–51. 10.1038/sj.cdd.4402081 17205078

[8] Kuter DJ. Treatment of chemotherapy-induced thrombocytopenia in patients with non-hematologic malignancies. Haematologica. 2022;107:1243–63. 10.3324/haematol.2021.279512 PMC9152964 35642485

[9] NCCN Clinical Practice Guidelines in Oncology (NCCN Guidelines®). Hematopoietic growth factors. 2024. Available at: https://www.nccn.org/professionals/physician_gls/pdf/growthfactors.pdf. Accessed September 7, 2025.

[10] Kuter DJ. The biology of thrombopoietin and thrombopoietin receptor agonists. Int J Hematol. 2013;98:10–23. 10.1007/s12185-013-1382-0 23821332

[11] Soff GA Miao Y Bendheim G. Romiplostim treatment of chemotherapy-induced thrombocytopenia. J Clin Oncol. 2019;37:2892–8. 10.1200/JCO.18.01931 PMC6823892 31545663

[12] Al-Samkari H Parnes AD Goodarzi K Weitzman JI Connors JM Kuter DJ. A multicenter study of romiplostim for chemotherapy-induced thrombocytopenia in solid tumors and hematologic malignancies. Haematologica. 2021;106:1148–57. 10.3324/haematol.2020.251900 PMC8018116 32499239

[13] Soff GA Al-Samkari H Leader A Eisen M Saad H. Romiplostim in chemotherapy-induced thrombocytopenia: a review of the literature. Cancer Med. 2024;13:e7429. 10.1002/cam4.7429 PMC11319220 39135303

[14] Wilkins CR Ortiz J Gilbert LJ. Romiplostim for chemotherapy-induced thrombocytopenia: efficacy and safety of extended use. Res Pract Thromb Haemost. 2022;6:e12701. 10.1002/rth2.12701 PMC9087952 35582038

[15] Zhao X Shan X Sui S Song Q Cheng M Zhao Y. Cancer treatment-induced thrombocytopenia: diagnosis, mechanisms and management. Front Immunol. 2025;16:1634688. 10.3389/fimmu.2025.1634688 PMC12500713 41063990

[16] Song AB Al-Samkari H. Chemotherapy-induced thrombocytopenia: modern diagnosis and treatment. Br J Haematol. 2025;206:1062–6. 10.1111/bjh.20037 PMC12140172 40040262

[17] Crawford J Denduluri N Patt D. Relative dose intensity of first-line chemotherapy and overall survival in patients with advanced non-small-cell lung cancer. Support Care Cancer. 2020;28:925–32. 10.1007/s00520-019-04875-1 PMC6954126 31172284

[18] Denduluri N Lyman GH Wang Y. Chemotherapy dose intensity and overall survival among patients with advanced breast or ovarian cancer. Clin Breast Cancer. 2018;18:380–6. 10.1016/j.clbc.2018.02.003 29622384

[19] Havrilesky LJ Reiner M Morrow PK Watson H Crawford J. A review of relative dose intensity and survival in patients with metastatic solid tumors. Crit Rev Oncol Hematol. 2015;93:203–10. 10.1016/j.critrevonc.2014.10.006 25459671

[20] Elting LS Rubenstein EB Martin CG. Incidence, cost, and outcomes of bleeding and chemotherapy dose modification among solid tumor patients with chemotherapy-induced thrombocytopenia. J Clin Oncol. 2001;19:1137–46. 10.1200/JCO.2001.19.4.1137 11181679

[21] Wilde MI Faulds D. Oprelvekin: a review of its pharmacology and therapeutic potential in chemotherapy-induced thrombocytopenia. BioDrugs. 1998;10:159–71. 10.2165/00063030-199810020-00006 18020592

[22] Vadhan-Raj S. Clinical experience with recombinant human thrombopoietin in chemotherapy-induced thrombocytopenia. Semin Hematol. 2000;37:28–34. 10.1053/shem.2000.7391 10831286

[23] Kuter DJ. New thrombopoietic growth factors. Blood. 2007;109:4607–16. 10.1182/blood-2006-10-019315 PMC1885525 17289815

[24] Cantor SB Elting LS Hudson DV Jr Rubenstein EB. Pharmacoeconomic analysis of oprelvekin (recombinant human interleukin-11) for secondary prophylaxis of thrombocytopenia in solid tumor patients receiving chemotherapy. Cancer. 2003;97:3099–106. 10.1002/cncr.11447 12784347

[25] Vadhan-Raj S Verschraegen CF Bueso-Ramos C. Recombinant human thrombopoietin attenuates carboplatin-induced severe thrombocytopenia and the need for platelet transfusions in patients with gynecologic cancer. Ann Intern Med. 2000;132:364–8. 10.7326/0003-4819-132-5-200003070-00005 10691586

[26] Geissler K Yin JA Ganser A. Prior and concurrent administration of recombinant human megakaryocyte growth and development factor in patients receiving consolidation chemotherapy for de novo acute myeloid leukemia–a randomized, placebo-controlled, double-blind safety and efficacy study. Ann Hematol. 2003;82:677–83. 10.1007/s00277-003-0737-7 14530872

[27] Basser RL O’Flaherty E Green M. Development of pancytopenia with neutralizing antibodies to thrombopoietin after multicycle chemotherapy supported by megakaryocyte growth and development factor. Blood. 2002;99:2599–602. 10.1182/blood.v99.7.2599 11895799

[28] Al-Samkari H Kuter DJ. Optimal use of thrombopoietin receptor agonists in immune thrombocytopenia. Ther Adv Hematol. 2019;10:2040620719841735. 10.1177/2040620719841735 PMC6460888 31007886

[29] Al-Samkari H Grace RF Kuter DJ. The role of romiplostim for pediatric patients with immune thrombocytopenia. Ther Adv Hematol. 2020;11:2040620720912992. 10.1177/2040620720912992 PMC7236573 32523658

[30] Townsley DM Scheinberg P Winkler T. Eltrombopag added to standard immunosuppression for aplastic anemia. N Engl J Med. 2017;376:1540–50. 10.1056/NEJMoa1613878 PMC5548296 28423296

[31] Nagrebetsky A Al-Samkari H Davis NM Kuter DJ Wiener-Kronish JP. Perioperative thrombocytopenia: evidence, evaluation, and emerging therapies. Br J Anaesth. 2019;122:19–31. 10.1016/j.bja.2018.09.010 30579402

[32] Soff GA Ray-Coquard I Rivera LJM. Systematic literature review and meta-analysis on use of thrombopoietic agents for chemotherapy-induced thrombocytopenia. PLoS One. 2022;17:e0257673. 10.1371/journal.pone.0257673 PMC9183450 35679540

[33] Qin S Wang Y Yao J. Hetrombopag for the management of chemotherapy-induced thrombocytopenia in patients with advanced solid tumors: a multicenter, randomized, double-blind, placebo-controlled, phase II study. Ther Adv Med Oncol. 2024;16:17588359241260985. 10.1177/17588359241260985 PMC11179448 38882443

[34] Kuter DJ. Novel therapies for immune thrombocytopenia. Br J Haematol. 2022;196:1311–28. 10.1111/bjh.17872 34611885

[35] Levchenkova OS Vorobieva VV Novikov VE Legonkova TI Volkova EN. Thrombopoietin receptor agonists in pharmacotherapy of pediatric immune thrombocytopenia. Res Results Pharmacol. 2023;9:49–59.

[36] Bussel JB Soff G Balduzzi A Cooper N Lawrence T Semple JW. A review of romiplostim mechanism of action and clinical applicability. Drug Des Devel Ther. 2021;15:2243–68. 10.2147/DDDT.S299591 PMC8165097 34079225

[37] Mytych DT Park JK Kim J. Assessment of romiplostim immunogenicity in adult patients in clinical trials and in a global postmarketing registry. Br J Haematol. 2020;190:923–32. 10.1111/bjh.16658 PMC7540503 32311075

[38] Wang B Nichol JL Sullivan JT. Pharmacodynamics and pharmacokinetics of AMG 531, a novel thrombopoietin receptor ligand. Clin Pharmacol Ther. 2004;76:628–38. 10.1016/j.clpt.2004.08.010 15592334

[39] Kuter DJ Bussel JB Newland A. Long-term treatment with romiplostim in patients with chronic immune thrombocytopenia: safety and efficacy. Br J Haematol. 2013;161:411–23. 10.1111/bjh.12260 23432528

[40] Romiplostim interactions. 2025. Available at: https://www.drugs.com/drug-interactions/romiplostim.html. Accessed September 22, 2025.

[41] Deng J Hu H Huang F. Comparative efficacy and safety of thrombopoietin receptor agonists in adults with thrombocytopenia: a systematic review and network meta-analysis of randomized controlled trial. Front Pharmacol. 2021;12:704093. 10.3389/fphar.2021.704093 PMC8355583 34393785

[42] Thrombosis, Hemostasis Group CSoHCMA. Chinese expert consensus on the clinical application of recombinant human thrombopoiein and thrombopoiein receptor agonist (2023). Zhonghua Xue Ye Xue Za Zhi. 2023;44:535–42. 10.3760/cma.j.issn.0253-2727.2023.07.002 PMC10509622 37749031

[43] Cantoni S Carpenedo M Mazzucconi MG. Alternate use of thrombopoietin receptor agonists in adult primary immune thrombocytopenia patients: a retrospective collaborative survey from Italian hematology centers. Am J Hematol. 2018;93:58–64. 10.1002/ajh.24935 28983953

